# Posttreatment stability following therapy using passive self-ligating brackets: extraction vs. nonextraction

**DOI:** 10.1007/s00056-023-00501-2

**Published:** 2023-10-17

**Authors:** Babak Sayahpour, Diana Lau, Sara Eslami, Sarah Buehling, Stefan Kopp, Abdolreza Jamilian, Sachin Chhatwani

**Affiliations:** 1 Department of Orthodontics, Center for Dentistry and Oral Medicine (Carolinum), Goethe University Frankfurt, Theodor-Stern-Kai 7, 60596 Frankfurt, Germany; 2 Dental Practice, Darmstädter Straße 44, 63225 Langen, Germany; 3 Department of Orthodontics, Center for Dentistry and Oral Medicine (Carolinum), Goethe University Frankfurt, Theodor-Stern-Kai 7, 60596 Frankfurt, Germany; 4 Department of Orthodontics, Center for Dentistry and Oral Medicine (Carolinum), Goethe University Frankfurt, Theodor-Stern-Kai 7, 60596 Frankfurt, Germany; 5 Department of Orthodontics, Center for Dentistry and Oral Medicine (Carolinum), Goethe University Frankfurt, Theodor-Stern-Kai 7, 60596 Frankfurt, Germany; 6 Department of Orthodontics, Dental School, Cranio-Maxillofacial Research Center, Tehran Islamic Azad University of Medical Sciences, Tehran, Iran; 7 City of London Dental School, University of Bolton, Bolton, UK; 8 Department of Orthodontics, Witten/Herdecke University, Alfred-Herrhausen-Straße 50, 58448 Witten, Germany

**Keywords:** Intercanine width, Extraction of mandibular premolars, Multibracket appliance, Arch width dimensions, Relapse, Interkanine Distanz, Extraktion von unteren Prämolaren, Multibracketapparatur, Maße der Bogenbreite, Rezidiv

## Abstract

**Purpose:**

This study aimed to evaluate the effects of lower premolar extraction on posttreatment stability one year following fixed orthodontic treatment with passive self-ligating brackets (Damon system, Ormco, Orange, CA, USA).

**Methods:**

All patients were treated with fixed orthodontic appliances using passive self-ligating brackets (Damon). For retention, removable Hawley retainers were used. Two groups of patients were included in the study. Each group consisted of 23 patients: group Ex consisted of 10 male and 13 female patients (13.4 ± 1.6 years old) with extraction of lower first premolars and group NonEx consisted of 11 male and 12 female patients (13.4 ± 3.9 years old) without dental extractions. The patients’ dental models and photographs were assessed at T0 (pretreatment), T1 (the end of active orthodontic treatment: 3.3 ± 1.0 years in the Ex and 2.3 ± 0.8 years in the NonEx group) and at T2 (1 year posttreatment). All lower casts were scanned and the following dental parameters were recorded and compared between the two groups: intercanine width (ICW), anterior arch width (AAW), intermolar width (IMW), Little’s irregularity index (LII) and gingival recessions.

**Results:**

An increase in ICW (group Ex: 1.20 ± 2.51 mm and group NonEx: 0.84 ± 1.48 mm) by the end of active treatment (T1; *P* < 0.05), as well as a relapse regarding the ICW (group Ex: −0.1 ± 0.47 mm and group NonEx: −67% ± 0.38 mm) one year post-treatment (T2) were recorded in the samples. Relapse in the non-extraction group was statistically and clinically significant, whereas ICW values remained relatively stable in the extraction group during the posttreatment period (T1–T2). The irregularity index decreased during treatment (group Ex: −8.79 ± 6.36 mm and group NonEx: −5.24 ± 2.99 mm) and relapsed in both groups (group Ex: 0.57 ± 90 mm and group NonEx: 0.27 ± 0.53). The rate of relapse in LII was correlated to the relapse rate of ICW. A reduction of IMW was recorded in the Ex group (−1.89 ± 1.82 mm) during active treatment (*P* < 0.05), which remained stable 1 year posttreatment. AAW increased in both groups (group Ex: 2.77 ± 1.77 mm and group NonEx: 1.77 ± 2.04 mm) throughout active treatment and remained stable at T2.

**Conclusion:**

Intergroup comparison revealed that ICW remained stable 1 year posttreatment in the Ex group, whereas high relapse of ICW was recorded in the NonEx group. Furthermore, risk of a relapse of LII appears to be higher in cases with a relapse of the ICW.

## Abstract (de)

**Ziel:**

Diese Studie hatte zum Ziel, die Auswirkungen der Extraktion der unteren Prämolaren auf die Nachbehandlungsstabilität ein Jahr nach kieferorthopädischer Multiband-Therapie mit passiven selbstligierenden Brackets (Damon-System, Ormco, Orange, CA, USA) zu untersuchen.

**Methoden:**

Alle Patienten wurden mit kieferorthopädischen Multibandapparaturen unter Verwendung von passiven selbstligierenden Brackets (Damon) behandelt. Zur Retention wurden herausnehmbare Hawley-Retainer eingesetzt. Zwei Patientengruppen zu je 23 Patienten wurden in die Studie aufgenommen: Gruppe Ex bestand aus 10 männlichen und 13 weiblichen Patienten (13,.4 ± 1,6 Jahre alt) mit Extraktion der unteren ersten Prämolaren, Gruppe NonEx aus 11 männlichen und 12 weiblichen Patienten (13,4 ± 3,9) ohne Zahnextraktionen. Die dentalen Modelle und Fotos der Patienten wurden zu T0 (vor der Behandlung), T1 (am Ende der aktiven kieferorthopädischen Behandlung: 3,3 ± 1.0 Jahre in der Gruppe Ex und 2,3 ± 0,8 Jahre in der Gruppe NonEx) und T2 (ein Jahr nach Entbänderung) analysiert und ausgewertet. Alle unteren Modelle wurden gescannt. Die nachfolgend aufgeführten zahnmedizinischen Parameter wurden erfasst und zwischen den beiden Gruppen verglichen: interkanine Distanz (ICW), anteriore Zahnbogenbreite (AAW), intermolare Distanz (IMW), Littles Irregularitätsindex (LII) und Gingivarezessionen.

**Ergebnisse:**

Festgestellt wurden eine Zunahme der ICW (Gruppe Ex: 1,20 ± 2,51 mm und Gruppe NonEx: 0,84 ± 1,48 mm) am Ende der aktiven Behandlung (T1; *p* < 0,05) sowie ein Rezidiv in Bezug auf die ICW (Gruppe Ex: −0,1 ± 0,47 mm und Gruppe NonEx: −67 % ± 0,38 mm) ein Jahr nach der Behandlung (T2) in den Stichproben. Das Rezidiv in der Nonextraktionsgruppe war statistisch und klinisch signifikant, während die ICW-Werte in der Extraktionsgruppe während der Nachbehandlungszeit (T1–T2) relativ stabil blieben. Der LII nahm während der Behandlung ab (Gruppe Ex: −8,79 ± 6,36 mm und Gruppe NonEx: −5,24 ± 2,99 mm) und zeigte in beiden Gruppen ein Rezidiv (Gruppe Ex: 0,57 ± 0,90 mm und Gruppe NonEx: 0,27 ± 0,53). Die Rate des Rezidivs im LII korrelierte mit der Rezidivrate der ICW. Eine Abnahme der IMW wurde in der Ex-Gruppe (−1,89 ± 1,82 mm) während der aktiven Behandlung verzeichnet (*p* < 0,05), sie zeigte sich ein Jahr nach der Behandlung stabil. Die AAW nahm in beiden Gruppen (Gruppe Ex: 2,77 ± 1,77 mm und Gruppe NonEx: 1,77 ± 2,04 mm) während der aktiven Behandlung zu und war zu T2 stabil.

**Schlussfolgerung:**

Der Intergruppenvergleich ergab, dass die ICW ein Jahr nach der Behandlung in der Ex-Gruppe stabil blieb, während ein hohes ICW-Rezidiv in der NonEx-Gruppe festgestellt wurde. Darüber hinaus scheint das Risiko eines LII-Rezidivs bei Patienten mit einem ICW-Rezidiv höher zu sein.

## Introduction

Posttreatment stability remains a major concern and a debated issue to this day. The maintenance of arch form and width (especially intercanine width) during active treatment is considered to play a major role in posttreatment stability [1].

Nevertheless, expansion of the dental arch in the posterior segment is proposed to be a suitable method to provide the required space for aligning and leveling in patients with crowding without increasing the intercanine width or the arch length through protrusion of the incisors, which would jeopardize posttreatment stability [2]. Several claims have been made by passive self-ligating bracket manufacturers (such as the Damon system, Damon Q, Ormco, CA, USA) on their ability to provide stable posterior expansion through physiological tooth movement [3]. However, strong clinical evidence supporting these claims is scarce. Most of the available studies on passive self-ligating brackets show a moderate increase in all transversal dental measurements including intercanine width [2, 4–7]. It is important to note that these studies were mainly performed on nonextraction cases with moderate to severe crowding. Nonextraction treatment of cases with severe crowding could lead to incisor protrusion and transversal expansion, in an amount that might decrease treatment stability regardless of the type of orthodontic appliance used. These studies very often evaluated the immediate posttreatment results and lack follow-up posttreatment evaluation. Their results are also controversial due to the use of different archwire types or sequences and retention protocols (fixed lingual retainers vs. removable retention appliances).

The decision for extraction vs. nonextraction therapy should not be based on the appliance (self-ligate vs. conventional brackets), but according to the individual criteria of each patient. Even though the extraction of permanent teeth has lost its popularity among orthodontists and has decreased by almost 20% [8], it is a well-stablished method of gaining space with justified indication in many patients [9]. Gaining space through extraction would theoretically minimize the unwanted increase of the dental arch width and length, reducing the risk of posttreatment relapse [10].

Another factor influencing posttreatment stability is a sufficient retention phase and the retention appliance. If the retention time is not adhered to, the risk of relapse is high, whereby the teeth return to their original position under the influence of the surrounding tissue that is still undergoing remodeling. Therefore, the new position of teeth should be kept stable for a sufficiently long retention time by suitable retention devices [11]. Bonded canine-to-canine retainers are effective long-term retainers, which very well maintain the anterior alignment and intercanine width after orthodontic treatment [12]. However, the use of fixed lingual retainers represents a confounding factor if evaluating posttreatment changes of the transversal dimension like the intercanine width and studies on posttreatment stability in the absence of fixed retainers are scarce.

Although previous studies have investigated the effects of extraction vs. nonextraction treatment, to our knowledge no study has compared the stability of the extraction vs. nonextraction modality using the Damon system.

Therefore, the aim of this study was to compare the posttreatment stability between nonextraction and extraction treatments utilizing passive self-ligating brackets (Damon system). The study aimed to test and reject the null hypothesis, which posited no difference in the posttreatment stability between the two treatment modalities.

## Materials and methods

### Sample size calculation

The sample size calculation was based on a study by Mueller [13]. In order to achieve a test power of 80% at an α significance level of 0.05, at least 21 patients per group were required to detect a mean difference greater than 1.5 mm. Thus, sample size was set at 23 patients per each group to counteract a possible dropout rate of 5%.

### Patients

The archive of the Orthodontic Department at Frankfurt University was searched for patients fulfilling the following inclusion criteria:

- Complete natural permanent dentition at the beginning of treatment,
- Skeletal class I malocclusion,
- Fully erupted lower canines at the beginning of treatment,
- Complete diagnostic records including preTx (T0), midTx (T1) and 1‑year (±½ year) postTx (T2) dental models and intraoral photographs with visible gingiva level,
- Patients undergoing fixed orthodontic therapy using Damon‑Q self-ligating brackets (Ormco, Orange, CA, USA) with standard values, 0.022-in slots and the following archwire sequence: 0.014 CuNiTi Damon (Ormco); 0.016 CuNiTi Damon (Ormco); 0.016 × 0.025 CuNiTi Damon (Ormco); 0.018 × 0.025 CuNiTi Damon (Ormco); 0.019 × 0.025 SS (Ormco), and
- Use of removable Hawley retention appliance during the first year posttreatment,

Exclusion criteria comprised

- Missing or supernumerary teeth, as well as prosthetic restorations,
- Systemic diseases, syndromes or skeletal dysgnathies, which require surgical intervention,
- Patients with poor compliance or oral hygiene which have led to early termination of the fixed orthodontic treatment,
- Gingival recessions prior to the beginning of treatment,
- Anterior cross bite,
- Previous removable, functional or fixed orthodontic treatment,
- Use of additional anchorage devices, expanders or fixed functional appliances.

### Data acquisition

In all, 46 patients fulfilled the inclusion criteria and were included in the study; 23 patients were treated without dental extractions and were allocated to the nonextraction (NonEx) group. Symmetric lower first premolar extractions (Ex) were performed in the other 23 patients and they were included in the Ex group.

**Fig. 1:**
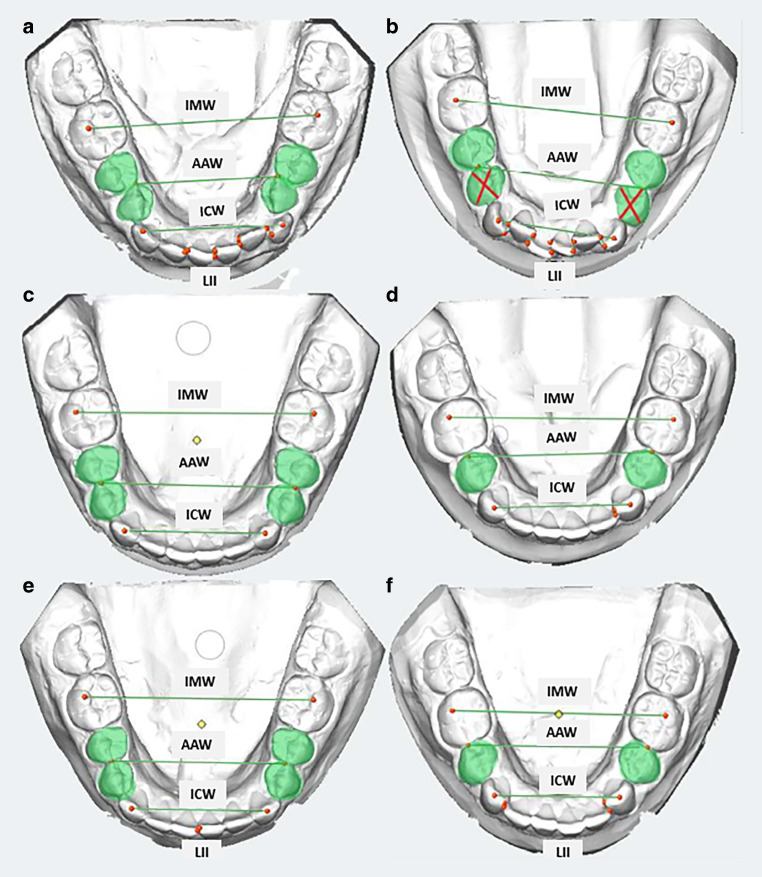
Three-dimensional dental imaging software screenshots of dental casts: **a** nonextraction case at T0, **b** extraction case at T0, **c** nonextraction case at T1, **d** extraction case at T1, **e** nonextraction case at T2, **f** extraction case at T2 (intercanine width (ICW), anterior arch width (AAW), intermolar width (IMW), Little’s irregularity index (LII)) Screenshot der dreidimensionalen zahnmedizinischen Bildgebungssoftware – Patientenmodell ohne Zahnextraktionen (NonEx) bei T0 **a** Nonextraktionsfall bei T0, **b** Extraktionsfall bei T0, **c** Nonextraktionsfall bei T1, **d** Extraktionsfall bei T1, **e** Nonextraktionsfall bei T2, **f** Extraktionsfall bei T2

Dental models and intraoral photographs of each patient were evaluated at three stages: at the beginning of the therapy (T0), immediately after debonding (T1), and 1 year after debonding (Fig. 1)).

All mandibular models were scanned with the orthoX model scanner (Dentaurum, Ispringen, Germany) and the following measurements were performed using the OnyxCeph^3TM^ software (Image Instruments, Chemnitz, Germany):

- Intercanine width (ICW): distance between the cusp tips of the canines.
- Anterior arch width (AAW): distance between the most buccal point of the approximal contact between the first and second premolar teeth.
- Intermolar width (IMW): distance between the tips of mesiobuccal cusps of the first molar teeth.
- Little’s irregularity index (LII): sum of the linear distances in the horizontal plane between the contact points of the anterior teeth including the mesial surface of the canines [14].

All measurements were done by one examiner and were repeated manually using a digital vernier caliper 1/100.

The photographs were evaluated for gingival recessions beyond the enamel–cement junction and were recorded by marking the yes or no boxes. Miller’s classification of the marginal tissue recessions was recorded in patients showing marginal recessions in the lower anterior segment (canine–canine) [15].

### Statistical analysis

The statistical analysis was done using the BiAS software for Windows (version 11.12, Epsilon Verlag, Darmstadt, Germany). Shapiro–Wilk test was used to check the variables for normal distribution. Since the data were not normally distributed, nonparametric tests were used. The Wilcoxon–Mann–Whitney U‑test was used for comparison between the Ex and NonEx groups. The comparisons between different time points within each group were performed using the Wilcoxon matched-pairs test.

The method error was assessed by repeating all of the manual measurements after 2 weeks by the same examiner using Dahlberg’s formula [16].

## Results

In all, 46 patients were included in the study (Table 1). Group Ex consisted of 10 male and 13 female patients (13.4 ± 1.6 years old) with extraction of lower first premolars and group NonEx consisted of 11 male and 12 female patients (13.4 ± 3.9 years old) without dental extractions. The patients in the Ex group showed on average a higher baseline LII (9.09 ± 6.43) than NonEx group (5.29 ± 2.97). Treatment duration was also on average longer in the Ex group (3.3 ± 1 years) compared to the NonEx group (2.3 ± 0.8 years). The results of the study model analyses are summarized in Table 2.

**Table 1:** Demographic characteristics of study groups Demografische Darstellung der Studiengruppen

Variable	NonEx Group	Ex Group	*P*-value
Number	23	23	NS
Gender female	12	13	NS
Gender male	11	10	NS
Age at T0 (mean ± SD)	13.4 ± 3.9	13.4 ± 1.6	NS

*T0* baseline, *T1* at the end of active treatment, *T2* 1 year posttreatment, *SD* standard deviation, *NS* not significant, *Ex* extraction, *NonEx* nonextraction

**Table 2:** Arch parameters in extraction (Ex) and nonextraction (NonEx) groups at T0 (baseline), T1 (at the end of active treatment), and T2 (1 year posttreatment) and also differences between time intervals Bogenbreitenparameter in den Gruppen Ex und NonEx bei T0 (Ausgangszustand), T1 (am Ende der aktiven Behandlung) und T2 (1 Jahr nach der Behandlung) sowie Unterschiede zwischen den Untersuchungszeitpunkten

Measurement	Group	T0 Mean (SD)	T1 Mean (SD)	T2 Mean (SD)	T0–T1 Mean (SD)	*P*-value	T1–T2 Mean (SD)	*P*-value	T0–T2 Mean (SD)	*P*-value
ICW	Ex	26.61 (2.91)	27.80 (1.46)	27.70 (1.57)	1.20 (2.51)	< 0.05*	−0.1 (0.47)	0.93 (NS)	1.10 (2.27)	< 0.05*
	NonEx	25.65 (1.80)	26.49 (1.12)	25.81 (1.31)	0.84 (1.48)	< 0.01**	−0.67 (0.38)	< 0.001***	0.17 (1.46)	0.12 (NS)
AAW	Ex	35.87 (2.93)	38.64 (1.90)	38.31 (1.81)	2.77 (2.23)	< 0.001***	−0.33 (0.82)	0.7 (NS)	2.45 (1.93)	< 0.001***
	NonEx	34.99 (2.44)	36.77 (1.66)	36.51 (1.75)	1.77 (2.04)	< 0.001***	0.25 (0.62)	0.07 (NS)	1.52 (1.67)	< 0.001***
IMW	Ex	48.27 (2.60)	46.38 (2.38)	46.38 (2.27)	−1.89 (1.82)	< 0.001***	0.0 (0.72)	0.09 (NS)	−1.89 (1.58)	< 0.001***
	NonEx	47.27 (1.82)	47.50 (2.06)	47.34 (2.01)	0.23 (1.25)	0.77 (NS)	−0.16 (0.70)	0.7 (NS)	0.07 (1.31)	0.7 (NS)
LII	Ex	9.09 (6.43)	0.30 (0.70)	0.87 (1.18)	−8.79 (6.36)	< 0.001***	0.57 (0.90)	< 0.01**	−8.22 (6.24)	< 0.001***
	NonEx	5.29 (2.97)	0.05 (0.17)	0.32 (0.59)	−5.24 (2.99)	< 0.001***	0.27 (0.53)	< 0.05*	−4.97 (3.04)	< 0.001***

*NS* not significant,* SD* standard deviation, *NS* not significant, *LII* Little’s irregularity index, *ICW* intercanine width, *AAW* anterior arch width, *IMW* intermolar width,* Ex* extraction, *NonEx* nonextraction

* low significance, ** significant, *** highly significant

The Dahlberg error was measured at 0.01 mm for ICW value. The intraexaminer evaluations showed minimal casual errors and absence of significant systematic errors.

### Intercanine width

A statistically significant increase in intercanine width (ICW) during active treatment (T0–T1) was noted in both groups (group Ex: 1.20 ± 2.51 mm and group NonEx: 0.84 ± 1.48 mm; Table 2). The ICW decreased posttreatment (T1–T2) in both groups (group Ex: −0.1 ± 0.47 mm and group NonEx: −0.67 ± 0.38 mm); however, this decrease was only statistically significant in the NonEx group (*P* < 0.001). Thus, in the NonEx group a significantly higher relapse was recorded and the values of intercanine width returned almost to the initial values 1 year posttreatment in this group (25.65 ± 1.80 mm at T0 and 25.81 ± 1.31 mm at T2).

Comparison between baseline and posttreatment CW measurements (T0–T2) revealed a statistically significant increase in the Ex group (1.10 ± 2.27 mm, *P* < 0.05), which is in contrast to the group NonEx (0.17 ± 1.46 mm, *P* = 0.12).

### Anterior arch width

The anterior arch width (AAW) values increased significantly during active treatment (T0–T1) in both groups (group Ex: 2.77 ± 2.23 mm and group NonEx: 1.77 ± 2.04 mm; *P* < 0.001). The anterior arch width remained relatively stable 1 year posttreatment and the changes in AAW between T1–T2 were not significant in the two groups (group Ex: −0.33 ± 0.82 mm, *P* = 0.7 and group NonEx: 0.25 ± 0.62 mm, *P* = 0.07).

### Intermolar width

Statistically significant changes were recorded in group Ex regarding the intermolar width (IMW) value. In this group, IMW decreased by −1.89 ± 1.8 2 mm from T0–T1 (*P* < 0.001) and remained stable during the posttreatment phase (T1–T2). The changes in the IMW value in the NonEx group (0.23 ± 1.25 mm during T0–T1 and −0.16 ± 0.70 mm during T1–T2) were not statistically significant between all investigation time points.

### Little’s irregularity index

The reduction in Little’s irregularity index (LII) measurements was statistically significant in both groups for the T0–T1 interval (*P* < 0.001). The reduction in Ex group (−8.79 ± 6.36 mm) was significantly higher than in the NonEx group (−5.24 ± 2.99 mm), which could be due to the higher baseline values of LII in the Ex group. A statistically significant and similar amount of relapse was noted in both groups (Ex group: 0.57 ± 0.90 mm, *P* < 0.01 and NonEx group: 0.27 ± 0.53 mm, *P* < 0.05) between T1 and T2. Nevertheless, the initial values of LII were not reached and both groups showed significantly lower LII values 1 year posttreatment in comparison with baseline measurements (*P* < 0.001).

### Concomitant relapse in ICW and LII measurement

Considering the whole patient collective revealed a relatively high risk of LII relapse of 13:5 (2.6) in case of concomitant ICW relapse. In cases without ICW relapse, a lower risk of LII relapse of 16:12 (1.3) was observed. The calculated odds ratio equals 1.95, meaning that the risk of LII relapse in the presence of a relapse of ICW was almost twice as high as that in the absence of ICW relapse.

### Gingival recession

None of the patients showed gingival recession in the lower anterior segment at T0. However, 3 patients in the Ex group and 5 patients in the NonEx group developed anterior gingival recession during active treatment (T0–T1). The gingival recession remained stable in these patients 1 year posttreatment. In addition, 1 patient in the Ex group and 1 patient in the NonEx group developed gingival recessions between T1 and T2. None of the patients showed a recession higher than class I according to Miller’s classification or the loss of interdental soft tissue.

## Discussion

The present study investigated posttreatment stability of extraction (Ex) vs. nonextraction (NonEx) treatment using passive self-ligating brackets (Damon system).

The Damon philosophy claims to resolve dental arch space deficiency by providing stable posterior arch expansion without compromising the perioral muscles [6]. Several studies have evaluated the effects of the Damon system (Damon, Ormco, Orange, CA, USA) on the arch parameters at the end of active treatment. Although the arch widening potential of the Damon system has been consistently shown by previous studies [2, 4–7], the increase in the transversal dimension was reported to happen throughout the arch (including the canine and premolar regions) and was mainly due to the broader form of the Damon archwires [5]. These results are partly in agreement with those of our study. We also reported an increase in ICW and AAW in both groups at T1, but an increase in IMW was not shown in our study. On the contrary, the IMW was even reduced in the Ex group at T1. This reduction could be explained by mesial movement of the molars towards the narrower part of the arch and by overall arch constriction due to premolar extraction in the Ex group. The same explanation applies to the increase of ICW in the Ex group: distal movement of the canines into the extraction site (broader part of the arch) leads to an increase in ICW at T1. In contrast to similar studies on NonEx treatment using the Damon system, the IMW remained unchanged in the NonEx group in our study. This was partly due to our archwire sequence. Although we have used the Damon arch form in the initial archwires, the last archwire used in our patients was an individualized stainless steel (SS) 0.019 × 0.025 archwire, which was planned to restore the patients individual arch form. The other reason for a less significant increase of IMW in the NonEx group is the exclusion of patients with severe crowding from the NonEx group. The majority of the studies on Damon system opted for a NonEx treatment and combined patients with moderate and severe crowding in the same group. The lower degree of expansion in our study at T1 may be explained by the lower degree of crowding in our NonEx group.

It is important to consider that most of the studies performed their measurements immediately at the end of active treatment (T1) and lack follow-up data on posttreatment stability of the Damon system. Thus, we also evaluated the stability of these parameters 1‑year posttreatment (T2). Furthermore, only a few studies have reported the posttreatment stability of the Damon system on the transversal dimension [2, 5, 17, 18]. Differences in the retention protocols should be considered if interpreting the stability results. The use of fixed lingual retainers is an effective method of preserving ICW and would explain the lack of relapse in ICW at T2 in the study by Lucchese et al. [5]. The authors also reported a tendency for posttreatment arch constriction especially in the premolar region, which could be due to the lack of compliance wearing a Hawley removable plate as an additional retention method [5]. In contrast to the study of Lucchese et al. [5], AAW and IMW remained relatively stable in both groups in our study during the T1–T2 interval. This may result from the difference in our archwire sequence, as we opted for individualized SS working archwires adapted to the patients’ initial archform, intending to decrease the degree of expansion and subsequent relapse at T2.

Our results regarding IMW and AAW are partly in agreement with those of Basciftci et al. [17], as they used a similar archwire sequence as ours. The high relapse tendency regarding ICW and LII at T2 in the NonEx group is largely due to not placing a fixed lingual retainer in our study.

Atik et al. [18] compared the transversal dimension stability of Damon system vs. a combination of Quadhelix and conventional brackets in patients with a transverse deficiency undergoing nonextraction treatment. They reported a significant relapse in ICW as well as interpremolar width in both of their groups. The authors suggested that the relapse was due to their retention protocol (only a removable Hawley plate), which is also in agreement with our results.

A similar study by Willeit et al. [2] showed a significant relapse in interpremolar width 1 year posttreatment, whereas ICW and IMW remained stable posttreatment. Their retention protocol consisted solely of fixed lingual retainer (no Essix or Hawley removable plate were used). The strength of the study by Willeit et al. is their long-term follow-up (6 years). They suggested that the relapse occurred mainly in the first year posttreatment and afterwards a plateau was reached [2]. However, the named studies only included nonextraction treatment and lacked information about the severity of the initial crowding or space deficiency in their samples, which might additionally explain the lack of agreement in their results with those of our study.

It is interesting to note that the high degree of relapse in the ICW in our study was only noted in the NonEx group, although the same retention protocol (removable Hawley plate) was used in both groups. Our results suggest that the role of fixed retention becomes even more critical following NonEx treatment, as a higher relapse is to be expected in ICW. The posttreatment stability of AAW in both groups suggest the efficiency of the Hawley plate in maintaining the posterior transversal dimension. Therefore, a combination of both retention devices can be recommended especially in patients undergoing NonEx treatment using the Damon system.

Comparing the stability of Ex vs. NonEx treatment, we found a greater reduction of LII in the Ex group. The greater reduction of LII in the Ex group can be explained by the higher baseline LII values in this group. Both groups showed a significant relapse one year posttreatment. This is relapse was higher in the NonEx group, which correlated with the more significant relapse of the ICW in this group at T2. Our results are in agreement with those of similar studies, using diverse bracket system [9, 19–22]. The use of the Damon system in our study did not seem to affect the posttreatment stability in regards to LII.

Cortin et al. [9] reported a greater long-term decrease of IMW in patients after dental extractions. In our study, IMW was reduced in the Ex group during active treatment and remained stable throughout the posttreatment phase. However, we retained the posterior transversal dimension using a Hawley appliance and investigated a shorter follow-up period of just one year, which is an important limitation of our study.

The study’s retrospective design certainly must be considered a limitation. Therefore, prospective studies with longer follow-up periods are required to further investigate the effects and stability of Ex treatment vs. NonEx using passive self-ligating brackets such as Damon system.

## Conclusion

Intercanine width (ICW) and anterior arch width (AAW) values significantly increased during active treatment in both the extraction (Ex) and nonextraction (NonEx) groups. A high relapse of ICW was recorded in the NonEx group, which correlated to the relapse in Little’s irregularity index (LII) measurements. Our results suggest higher stability in the Ex group regarding the ICW 1 year posttreatment. Therefore, the use of the self-ligating system in our study did not lead to more stable expansion.

Intermolar width (IMW) remained stable during all intervals in the NonEx group in contrast to the Ex group, which showed a significant reduction of the IMW value between T0 and T1. This reduction remained stable 1 year posttreatment. This finding highlights the role of the archwire form vs. the ligation method with regard to the arch widening effect of a self-ligating system.

## References

[1] Shirazi S, Kachoei M, Shahvaghar-Asl N, Shirazi S, Sharghi R (2016) Arch width changes in patients with class II division 1 malocclusion treated with maxillary first premolar extraction and non-extraction method. J Clin Exp Dent 8(4):e403–e408. 10.4317/jced.52840 27703608 10.4317/jced.52840 PMC5045687

[2] Willeit FJ, Cremonini F, Willeit P, Ramina F, Cappelletti M, Spedicato GA, Lombardo L (2022) Stability of transverse dental arch dimension with passive self-ligating brackets: a 6-year follow-up study. Prog Orthod 23(1):34. 10.1186/s40510-022-00414-7 35718801 10.1186/s40510-022-00414-7 PMC9207026

[3] Yang X, Xue C, He Y, Zhao M, Luo M, Wang P, Bai D (2018) Transversal changes, space closure, and efficiency of conventional and self-ligating appliances. J Orofac Orthop 79(1):1–10. 10.1007/s00056-017-0110-4 29101414 10.1007/s00056-017-0110-4

[4] Atik E, Ciğer S (2014) An assessment of conventional and self-ligating brackets in class I maxillary constriction patients. Angle Orthod 84(4):615–622. 10.2319/093013-712.1 24423203 10.2319/093013-712.1 PMC8650438

[5] Lucchese A, Manuelli M, Albertini P, Ghislanzoni LH (2019) Transverse and torque dental changes after passive self-ligating fixed therapy: a two-year follow-up study. Am J Orthod Dentofacial Orthop 156(1):94–103. 10.1016/j.ajodo.2018.08.019 31256848 10.1016/j.ajodo.2018.08.019

[6] Vajaria R, BeGole E, Kusnoto B, Galang MT, Obrez A (2011) Evaluation of incisor position and dental transverse dimensional changes using the Damon system. Angle Orthod 81(4):647–652. 10.2319/071910-420.1 21446870 10.2319/071910-420.1 PMC8919756

[7] Pandis N, Polychronopoulou A, Makou M, Eliades T (2010) Mandibular dental arch changes associated with treatment of crowding using self-ligating and conventional brackets. Eur J Orthod 32(3):248–253. 10.1093/ejo/cjp123 19959610 10.1093/ejo/cjp123

[8] Dardengo Cde S, Fernandes LQ, Capelli Júnior J (2016) Frequency of orthodontic extraction. Dental Press J Orthod 21:54–59. 10.1590/2177-6709.21.1.054-059.oar 27007762 10.1590/2177-6709.21.1.054-059.oar PMC4816586

[9] Cotrin P, Gambardela-Tkacz CM, Moura W, Iunes A, Janson G, Freitas MR, Freitas KMS (2020) Anterior tooth alignment and arch dimensions changes: 37-year follow-up in patients treated with and without premolar extraction. Am J Orthod Dentofacial Orthop 158(4):e5–e15. 10.1016/j.ajodo.2020.07.013 32843251 10.1016/j.ajodo.2020.07.013

[10] Gorucu-Coskuner H, Atik E, Kocadereli I (2017) Effects of three different orthodontic treatment methods on the stability of mandibular incisor alignment. J Clin Pediatr Dent 41(6):486–493. 10.17796/1053-4628-41.6.13 29087804 10.17796/1053-4628-41.6.13

[11] Padmos JAD, Fudalej PS, Renkema AM (2018) Epidemiologic study of orthodontic retention procedures. Am J Orthod Dentofacial Orthop 153(4):496–504. 10.1016/j.ajodo.2017.08.013 29602341 10.1016/j.ajodo.2017.08.013

[12] Renkema AM, Renkema A, Bronkhorst E, Katsaros C (2011) Long-term effectiveness of canine-to-canine bonded flexible spiral wire lingual retainers. Am J Orthod Dentofacial Orthop 139(5):614–621. 10.1016/j.ajodo.2009.06.041 21536204 10.1016/j.ajodo.2009.06.041

[13] Mueller UH (2017) Langzeitstabilität nach Straight-Wire Therapie in Abhängigkeit von der individuellen Protrusion der Unterkieferschneidezähne (https://scidok.sulb.uni-saarland.de/bitstream/20.500.11880/26813/1/Dissertation_MAller_U_29_01_2017_M_ohne_Lebenslauf.pdf)

[14] Little RM (1975) The irregularity index: a quantitative score of mandibular anterior alignment. Am J Orthod 68(5):554–563. 10.1016/0002-9416(75)90086-x 1059332 10.1016/0002-9416(75)90086-x

[15] Miller PD (2018) Miller classification of marginal tissue recession revisited after 35 years. Compend Contin Educ Dent 39(8):514–520 30188152

[16] Dahlberg G (1940) Statistical methods for medical and biological students. Br Med J 2(4158):358–359

[17] Basciftci FA, Akin M, Ileri Z, Bayram S (2014) Long-term stability of dentoalveolar, skeletal, and soft tissue changes after non-extraction treatment with a self-ligating system. Korean J Orthod 44(3):119–127. 10.4041/kjod.2014.44.3.119 24892025 10.4041/kjod.2014.44.3.119 PMC4040359

[18] Atik E, Taner T (2017) Stability comparison of two different dentoalveolar expansion treatment protocols. Dental Press J Orthod 22:75–82. 10.1590/2177-6709.22.5.075-082.oar 29160347 10.1590/2177-6709.22.5.075-082.oar PMC5730139

[19] Artun J, Garol JD, Little RM (1996) Long-term stability of mandibular incisors following successful treatment of class II, division 1, malocclusions. Angle Orthod 66:229–238. 10.1043/0003-3219(1996)066%3C0229:LTSOMI%3E2.3.CO;2 8805919 10.1043/0003-3219(1996)066<0229:LTSOMI>2.3.CO;2

[20] Dyer KC, Vaden JL, Harris EF (2012) Relapse revisited—again. Am J Orthod Dentofacial Orthop 142:221–227. 10.1016/j.ajodo.2012.03.030 22858332 10.1016/j.ajodo.2012.03.030

[21] Paquette DE, Beattie JR, Johnston LE Jr (1992) A long-term comparison of non-extraction and premolar extraction edgewise therapy in “borderline” class II patients. Am J Orthod Dentofacial Orthop 102:1–14. 10.1016/0889-5406(92)70009-Y 1626523 10.1016/0889-5406(92)70009-Y

[22] Luppanapornlarp S, Johnston LE Jr (1993) The effects of premolar- extraction: a long-term comparison of outcomes in “clear-cut” extraction and non-extraction class II patients. Angle Orthod 63:257–272. 10.1043/0003-3219(1993)063%3C0257:TEOPAL%3E2.0.CO;2 8297050 10.1043/0003-3219(1993)063<0257:TEOPAL>2.0.CO;2

